# Formamidinium Incorporates into Rb‐based Non‐Perovskite Phases in Solar Cell Formulations

**DOI:** 10.1002/anie.202416938

**Published:** 2024-11-14

**Authors:** Ummugulsum Gunes, Michael A. Hope, Yuxuan Zhang, Likai Zheng, Lukas Pfeifer, Michael Grätzel, Lyndon Emsley

**Affiliations:** ^1^ Institut des Sciences et Ingenierie Chimiques École Polytechnique Fedérale de Lausanne (EPFL) CH-1015 Lausanne Switzerland; ^2^ Department of Chemistry University of Warwick Gibbet Hill Road Coventry CV4 7AL UK

**Keywords:** NMR Spectroscopy, Perovskite Phases, Phase Segregation

## Abstract

Organic‐inorganic hybrid perovskite materials, such as formamidinium lead iodide (FAPbI_3_), are among the most promising emerging photovoltaic materials. However, the spontaneous phase transition from the photoactive perovskite phase to an inactive non‐perovskite phase complicates the application of FAPbI_3_ in solar cells. To remedy this, alkali metal cations, most often Cs^+^, Rb^+^ or K^+^, are included during perovskite synthesis to stabilize the photoactive phase. The atomic‐level mechanisms of stabilization are complex. While Cs^+^ dopes directly into the perovskite lattice, Rb^+^ does not, but instead forms an additional non‐perovskite phase, and the mechanism by which Rb confers increased stability remains unclear. Here, we use ^1^H−^87^Rb double resonance NMR experiments to show that FA^+^ incorporates into the Rb‐based non‐perovskite phases (FA_*y*_Rb_1‐*y*_Pb_2_Br_5_ and δ‐FA_*y*_Rb_1‐*y*_PbI_3_) for both bromide and iodide perovskite formulations. This is demonstrated by changes in the ^1^H and ^87^Rb chemical shifts, ^1^H−^87^Rb heteronuclear correlation spectra, and ^87^Rb{^1^H} REDOR spectra. Simulation of the REDOR dephasing curves suggests up to ~60 % FA^+^ incorporation into the inorganic Rb‐based phase for the bromide system. In light of these results, we hypothesize that the substitution of FA^+^ into the non‐perovskite phase may contribute to the greater stability conferred by Rb salts in the synthesis of FA‐based perovskites.

## Graphical abstract

We determine the speciation in mechanosynthesized formamidinium (FA^+^) and rubidium (Rb^+^)‐containing lead‐halide perovskite formulations by NMR. We discover that a secondary non‐perovskite Rb‐based phase incorporates up to 60 % formamidinium.

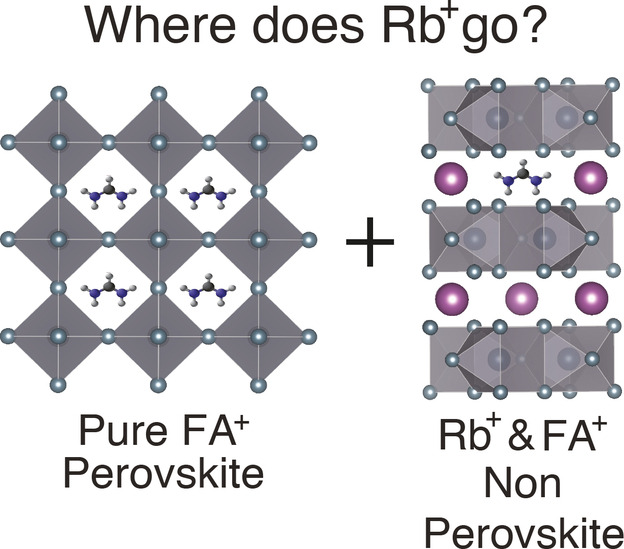

## Introduction

**Figure 1:**
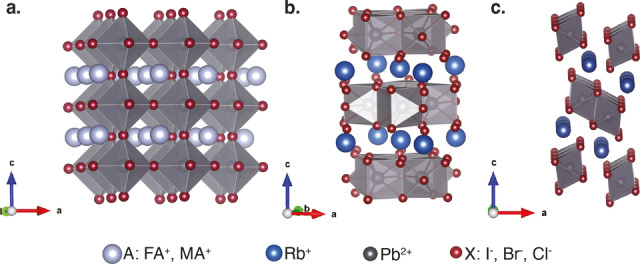
Crystal structure of a) a photoactive cubic perovskite phase (*Pm* $\bar{3}$ *m*), b) RbPb_2_Br_5_ (*I*4/*mcm*), and c) δ‐RbPbI_3_ (*Pnma*). Based on CCDC entry 1474200, ICSD entries 151990, and 161482.[^45^, ^46^, ^47^]

Perovskite solar cells are a pivotal emerging photovoltaic technology with remarkable optoelectronic properties including high optical absorption coefficients and long charge carrier length.[^1^, ^2^, ^3^, ^4^, ^5^, ^6^] Their power conversion efficiencies (PCEs) have now exceeded 26 %,^[7]^ which is similar to the efficiency of conventional silicon solar cells. Lead‐halide perovskite materials have the general formula APbX_3_ in which A^+^ is a small organic cation such as formamidinium (FA^+^) or methylammonium (MA^+^), an inorganic cation such as cesium (Cs^+^), or a mixture of cations, and X^−^ is a halide or mixture of halides (I^−^, Br^−^, Cl^−^). The perovskite structure consists of corner‐sharing [PbX_6_] octahedra with the A‐site cation fitting into the cuboctahedral voids (Figure 1a).^[8]^

The first perovskite solar cells used methylammonium lead iodide (MAPbI_3_) as the main absorber material.[^9^, ^10^] However, its limited light‐harvesting properties and its instability under operational conditions led to an intense search for new absorber materials.[^11^, ^12^, ^13^] Today, formamidinium lead iodide (FAPbI_3_) is the most popular absorber layer due to its near‐ideal band gap of 1.48 eV.[^14^, ^15^, ^16^, ^17^, ^18^] However, the key weakness of FAPbI_3_ is its thermodynamic instability at room temperature due to the large size of the FA^+^ cation. It transforms spontaneously from the photoactive cubic perovskite phase (α‐FAPbI_3_) to an undesirable non‐photoactive hexagonal phase (δ‐FAPbI_3_).[^15^, ^19^] There have been a number of approaches to prevent this phase transformation by adding various salts to the FAPbI_3_ precursor solutions, including organic cations such as MA^+^ and GUA^+^ (guanidinium), alkali metal ions (Cs^+^, Rb^+^_,_ or K^+^), or lanthanides.[^20^, ^21^, ^22^, ^23^, ^24^, ^25^] A variety of perovskite compositions have been reported with double (generally FA/MA or Cs/FA), triple (usually Cs/FA/MA), or quadruple (Rb/Cs/FA/MA) cation combinations.[^26^, ^27^, ^28^, ^29^, ^30^] However, despite reports that these formulations produce pure perovskite phases, there is evidence that the smaller inorganic cations Rb^+^ and K^+^ cannot substitute into hybrid perovskites based on organic cations.[^31^, ^32^]

Indeed, the phase stability of the perovskite crystal structures is often guided by the Goldschmidt tolerance factor which is defined as t = (r_A_ + r_X_)/[√2(r_B_ + r_X_)], where r_A_, r_B_, and r_X_ are the ionic radii for the A^+^, B^2+^, and X^−^ ions, respectively.[^33^, ^34^] To obtain a stable cubic perovskite structure, *t* should be in the range 0.8–1.[^35^, ^36^] Therefore, the size of the A‐site cation plays a major role in the phase stability.^[37]^ We also note that the A‐site cation plays an indirect role in the optoelectronic properties of the solar cells as it can change the band gap by changing the tilt angle between the Pb^2+^ and X^−^ species.[^34^, ^36^]

Solid‐state NMR spectroscopy is ideally suited to study the incorporation of dopants in halide perovskites, as well as various other atomic‐level structural and dynamic features, as an element‐specific probe of local structure and atomic proximities.[^38^, ^39^, ^40^, ^41^, ^42^] In particular, Kubicki et al. used ^87^Rb NMR to show that Rb^+^ did not dope into CsFAMA or FAMA‐based iodide or mixed iodide–bromide perovskite systems but instead segregated into a Rb or Rb/Cs inorganic non‐perovskite lead‐halide phase (Figure 1b,c).^[31]^ Even though Rb^+^ is not incorporated into the perovskite lattice, there is a clear increase in PCE with the addition of RbI to the precursor solution.[^28^, ^43^, ^44^] It has been hypothesized that this arises from surface passivation by the more stable inorganic non‐perovskite phases, but to date, little is known of these phases.

Here, we use solid‐state NMR spectroscopy to study the formation of Rb‐based non‐perovskite phases formed in mechanosynthesized “Rb_*x*_FA_1−*x*_PbBr_3_” and “Rb_*x*_FA_1−*x*_PbI_3_” perovskite formulations that include small amounts of Rb^+^. Surprisingly, ^1^H−^87^Rb double resonance experiments show that formamidinium can substitute into the non‐perovskite phases (FA_*y*_Rb_1−*y*_Pb_2_Br_5_ and δ‐FA_*y*_Rb_1−*y*_PbI_3_), up to ca. 60 % in the bromide and ca. 15 % in the iodide case.

## Results and Discussion

### “Rb_x_FA_1−x_PbBr_3_” Formulations

The “Rb_*x*_FA_1−*x*_PbBr_3_” formulations (*x*: 0.01, 0.03 and 0.10) were synthesized by mechanochemical synthesis, followed by powder X‐ray diffraction (XRD) measurements. Note that throughout, the composition of the formulation is shown in quotation marks and *x* refers to the amount of Rb in the formulation; this does not denote the composition of a single pure phase. The XRD patterns (Figure S1) show that none of the samples form a single pure Rb_*x*_FA_1−*x*_PbBr_3_ phase, but instead the sample segregates into two components: the α‐FAPbBr_3_ perovskite phase and a non‐perovskite phase that matches RbPb_2_Br_5_ (structure shown in Figure 1b).^[46]^

**Figure 2:**
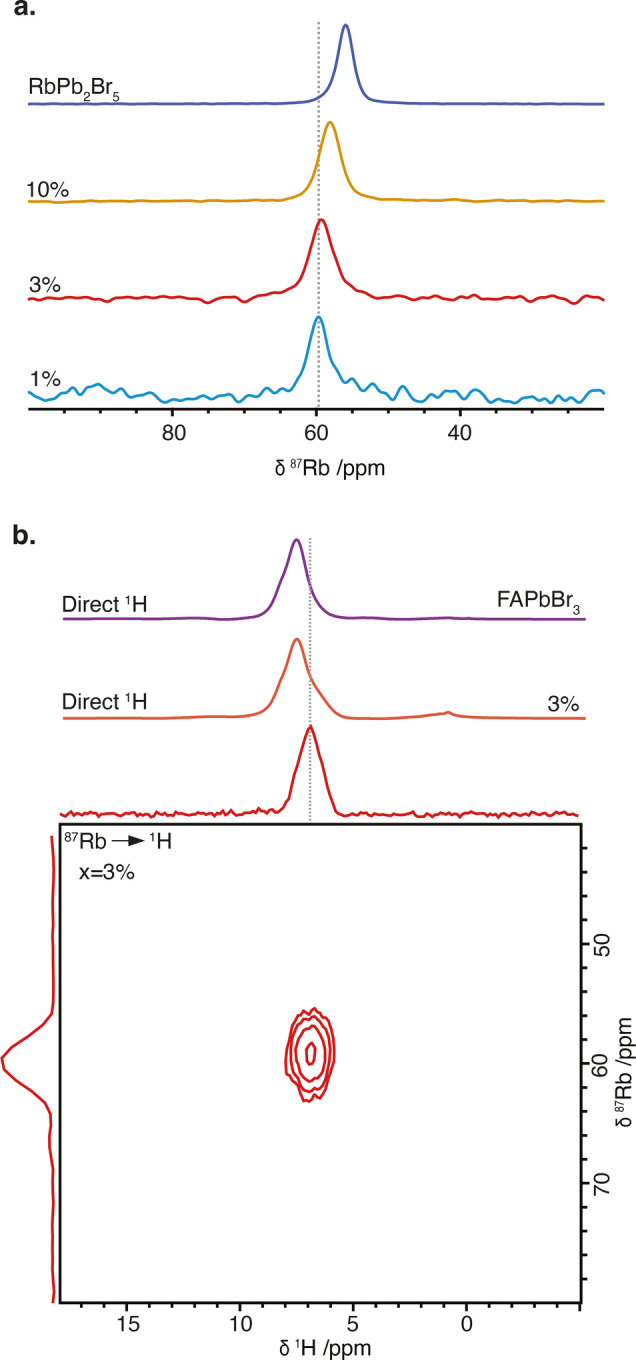
a) ^87^Rb NMR spectra of RbPb_2_Br_5_ and “Rb_*x*_FA_1−*x*_PbBr_3_” formulations (*x*: 0.01, 0.03 and 0.10) acquired with a central‐transition (CT) selective Hahn echo.^[50]^ b) ^87^Rb→^1^H HETCOR spectrum of the “Rb_0.03_FA_0.97_PbBr_3_” composition, the projection onto the ^1^H and ^87^Rb axes and the direct 1D ^1^H NMR spectra of “Rb_0.03_FA_0.97_PbBr_3_” and FAPbBr_3_ samples. All spectra were recorded at room temperature and 20 kHz MAS. Experimental details are given in SI, together with a link to all the raw data.

^87^Rb NMR spectra were acquired for RbPb_2_Br_5_ and all the “Rb_*x*_FA_1−*x*_PbBr_3_” samples to confirm the Rb speciation. As shown in Figure 2a, the spectrum of pure RbPb_2_Br_5_ is centered at 56.0 ppm. For the “Rb_*x*_FA_1−*x*_PbBr_3_” samples, the isotropic chemical shift varies systematically with the Rb^+^ content (δ ^87^Rb = 60.1, 59.4 and 58.4 ppm for *x* = 0.01, 0.03, and 0.10, respectively). In addition, all the samples have broader line widths than for pure RbPb_2_Br_5_, indicating a greater distribution of environments.

Figure 2b and Figure S2 show the directly acquired ^1^H NMR spectra for the mixed‐cation “Rb_*x*_FA_1−*x*_PbBr_3_” samples. The samples show an asymmetric ^1^H peak centered around 7.7 ppm, as expected for FAPbBr_3_ perovskites.[^48^, ^49^] This suggests that the majority of the FA^+^ cations are present in the perovskite phase.

^87^Rb→^1^H cross polarization (CP) experiments were then performed to identify any correlations between the Rb^+^ and FA^+^ cations. A clear correlation peak is observed at δ ^1^H = 6.8 ppm and δ ^87^Rb = 59.8 ppm between ^87^Rb and ^1^H nuclei in the ^87^Rb→^1^H heteronuclear correlation (HETCOR) spectrum of Rb_0.03_FA_0.97_PbBr_3_ (Figure 2b). The same signal is observed for the other two samples (Figure S2). Considering that FA^+^ is the only proton source in the materials, we conclude that there are FA^+^ protons in close proximity to rubidium atoms (i.e., <7 Å). However, the peak positions do not match the direct ^1^H spectra (Figure 2b). Since the ^87^Rb shifts are very similar to that of pure RbPb_2_Br_5_, the correlated 6.8 ppm ^1^H signals are assigned to a minority of FA^+^ cations incorporated in a FA_*y*_Rb_1−*y*_Pb_2_Br_5_ non‐perovskite phase (we will use *y* to refer to the proportion of FA^+^ in the non‐perovskite phase, which should not be confused with the Rb content in the overall formulation, *x*). Note that since this is a minority phase, it cannot be resolved from the major FAPbBr_3_ peak in the direct ^1^H spectra.

**Figure 3:**
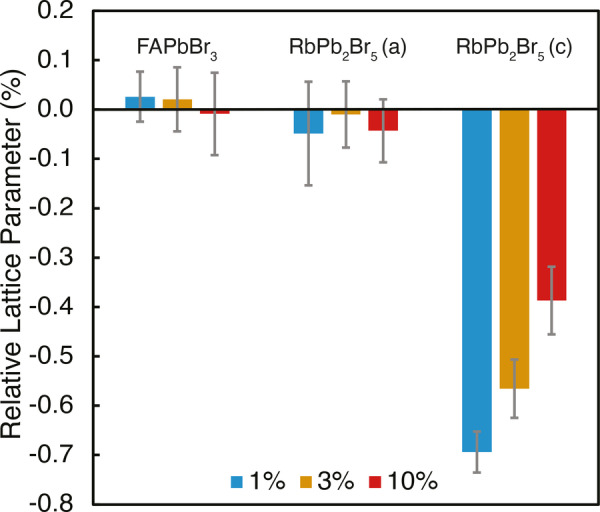
Relative lattice parameters measured for the cubic FAPbBr_3_ (*a*=*b*=*c*) and tetragonal FA_*y*_Rb_1−*y*_Pb_2_Br_5_ (*a*=*b*≠*c*) phases observed in the “Rb_*x*_FA_1−*x*_PbBr_3_” formulations (*x*=0.01, 0.03, and 0.10). The relative change is defined as $\left(\lambda_{mixed}-\lambda_{pure}\right)/\lambda_{pure}$ , where $\lambda_{mixed}$ is the lattice parameter in the mixed formulation and $\lambda_{pure}$ is the lattice parameter in FAPbBr_3_ or RbPb_2_Br_5_.

Further evidence for FA^+^ incorporation in a FA_*y*_Rb_1−*y*_Pb_2_Br_5_ phase comes from changes in the lattice parameters, as determined by Pawley refinement^[51]^ of the powder XRD patterns (Table S4). Figure 3 shows the relative change in the lattice parameters for the FAPbBr_3_ and RbPb_2_Br_5_‐like phases in the mixed “Rb_*x*_FA_1−*x*_PbBr_3_” formulations, with respect to the pure reference FAPbBr_3_ and RbPb_2_Br_5_ phases. There is no significant change in the FAPbBr_3_ lattice parameter, which is consistent with no incorporation of Rb^+^ in the perovskite phase. In contrast, we observe that the *c* lattice parameter for the RbPb_2_Br_5_‐like phase in the mixed samples is significantly different to that of pure RbPb_2_Br_5_, and changes systematically with the composition. The more Rb is included in the formulation, the larger the *c* lattice parameter, which strongly correlates with the measured ^87^Rb shifts (Figure S3). Together with the ^87^Rb→^1^H NMR data, this convincingly supports the formation of a mixed FA_*y*_Rb_1−*y*_Pb_2_Br_5_ phase. The change in the *c* lattice parameter is a consequence of the structure of RbPb_2_Br_5_, where layers of Rb^+^ cations are sandwiched between [Pb_2_Br_5_]^−^ layers along the *c* direction (Figure 1b).[^46^, ^52^] The contraction of the *c* lattice parameter, despite the larger nominal size of FA^+^ vs. Rb^+^,^[53]^ may be due to H‐bonding between FA^+^ and Br^−^.^[54]^

**Figure 4:**
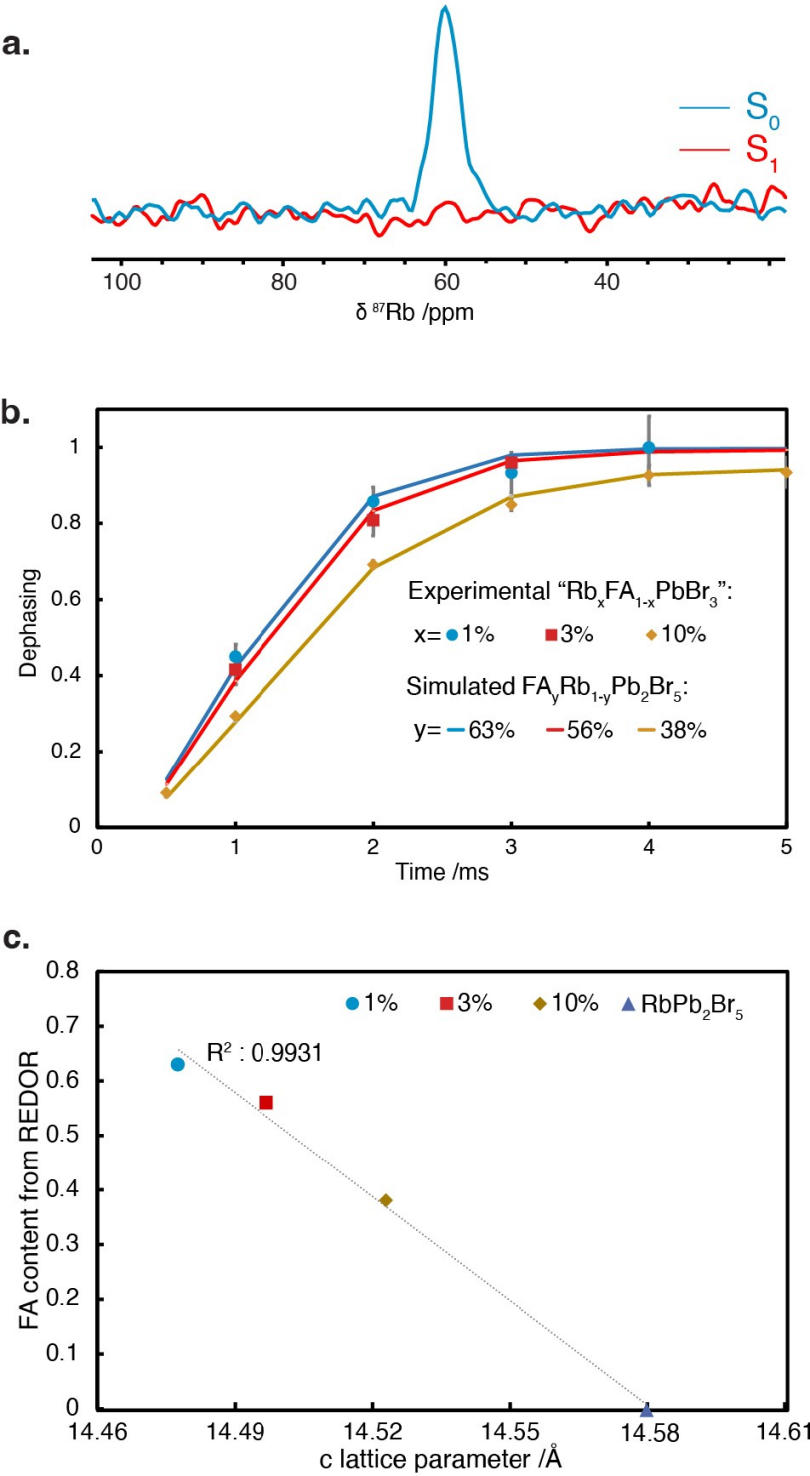
a) ^87^Rb{^1^H} REDOR spectra of the “Rb_0.01_FA_0.99_PbBr_3_” sample with (*S*_1_) and without (*S*_0_) ^1^H dipolar recoupling for 4 ms prior to acquisition. b) Relative ^87^Rb{^1^H} REDOR dephasing, (S_0_−S_1_)/S_0_, as a function of the recoupling time (markers) for the FA_*y*_Rb_1−*y*_Pb_2_Br_5_ phase in each “Rb_*x*_FA_1−*x*_PbBr_3_” sample, and simulated dephasing curves (lines) for different FA^+^ contents in FA_*y*_Rb_1−*y*_Pb_2_Br_5_. The error bars were obtained by dividing (S_0_−S_1_)/S_0_ by the signal to noise ratio (SNR) obtained from the respective spectra. c) Linear regression of the FA^+^ content of the mixtures obtained from the REDOR experiments against the *c* lattice parameter obtained from Pawley refinement. Experimental details are given in SI, together with a link to all the raw data.

Having demonstrated the formation of a mixed FA_*y*_Rb_1−*y*_Pb_2_Br_5_ phase, we used ^87^Rb{^1^H} Rotational Echo Double Resonance (REDOR) experiments to estimate the degree of substitution. Here, we measure the ^87^Rb spectrum with and without applying ^1^H recoupling in order to reintroduce the ^1^H−^87^Rb heteronuclear dipolar couplings.^[38]^ This leads to attenuation of the ^87^Rb signal as a function of the recoupling time (referred to as dephasing) that depends on the strength of the dipolar coupling, and hence the number of adjacent ^1^H nuclei and their distance from the ^87^Rb. The ^87^Rb{^1^H} REDOR spectrum of the “Rb_0.01_FA_0.99_PbBr_3_” sample (Figure 4a) shows complete dephasing of the FA_*y*_Rb_1−*y*_Pb_2_Br_5_^87^Rb signal with 4 ms of ^1^H recoupling. This indicates that all Rb^+^ cations are in close contact with FA^+^ protons (<ca. 7 Å), suggesting a high degree of FA^+^ incorporation.

Quantitative REDOR analysis can be achieved by measuring the degree of dephasing as a function of the recoupling time (Figure 4b). The observed REDOR dephasing is slower for the samples with lower FA^+^/Rb^+^ ratios (3 % and 10 % Rb), which indicates less incorporation of FA^+^ in the FA_*y*_Rb_1−*y*_Pb_2_Br_5_ phase. To estimate the amount of FA^+^ present in the non‐perovskite phase, we simulated REDOR dephasing curves as a function of FA^+^ content and fitted them to the experimental data (see the Supporting Information for details). The simulations successfully reproduce the experimental data, as shown in Figure 4b (solid lines). From this we determine compositions for the non‐perovskite phases of FA_0.63_Rb_0.37_Pb_2_Br_5_, FA_0.56_Rb_0.44_Pb_2_Br_5_ and FA_0.38_Rb_0.62_Pb_2_Br_5_ for the 1 %, 3 % and 10 % Rb samples, respectively. To corroborate these values, we plotted the FA^+^ concentrations determined by REDOR against the experimental *c* lattice parameters, which are sensitive to the composition (see above). Figure 4c shows a strong linear correlation, as expected from Vegard's law.^[55]^

In summary, we find that when Rb^+^ is included in the perovskite formulation (“Rb_*x*_FA_1−*x*_PbBr_3_”), together with FA^+^ it forms a non‐perovskite phase, FA_*y*_Rb_1−*y*_Pb_2_Br_5_ (See Supplementary Note 2 for the reaction chemical equation). The lower the Rb concentration in the formulation, the more FA^+^ is incorporated in this non‐perovskite phase, up to *y* ≈ 63 % for a Rb composition of *x* = 1 %.

### “Rb_x_FA_1−x_PbI_3_” Formulations

**Figure 5:**
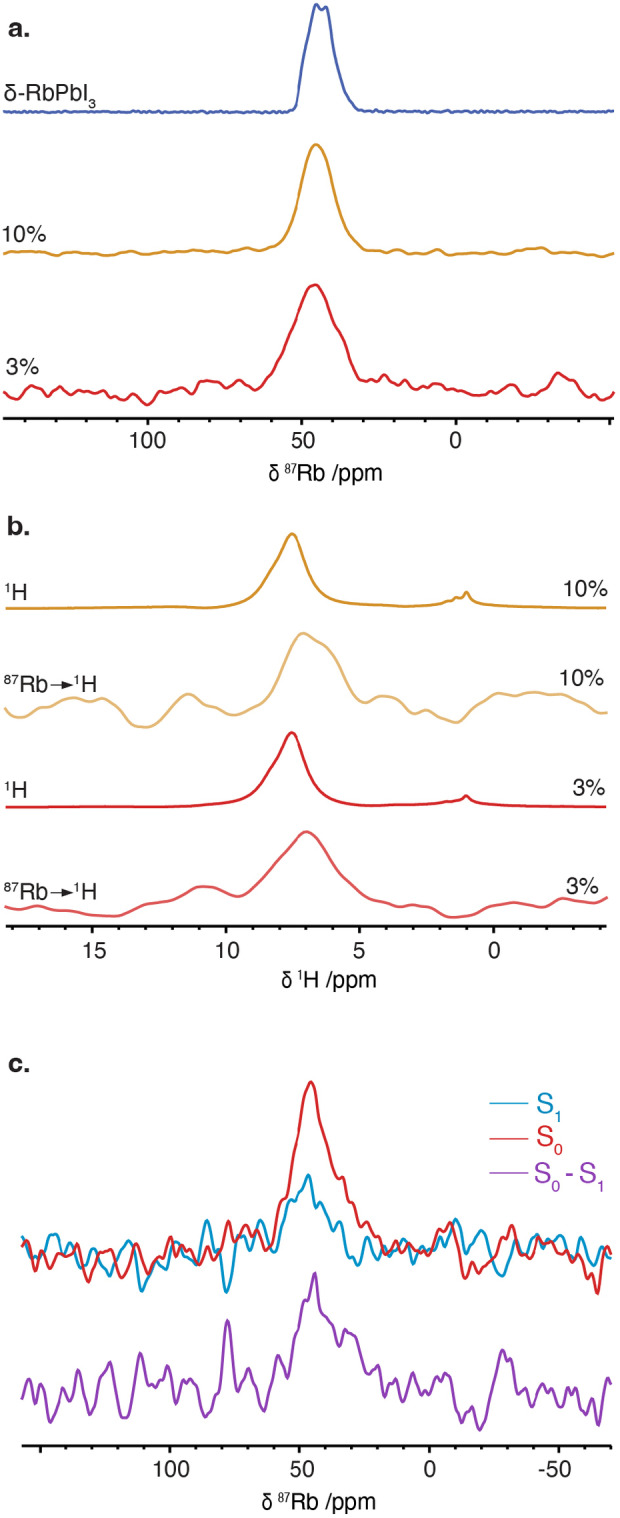
a) ^87^Rb NMR spectra of the δ‐RbPbI_3_ and “Rb_*x*_FA_1−*x*_PbI_3_” formulations (*x*: 0.03 and 0.10) acquired with a CT‐selective Hahn echo. b) Slice from the ^87^Rb→^1^H HETCOR spectrum of “Rb_0.03_FA_0.97_PbI_3_” composition, ^87^Rb→^1^H cross polarization spectrum of the “Rb_0.10_FA_0.90_PbI_3_” sample and direct ^1^H spectrum of “Rb_*x*_FA_1−*x*_PbI_3_” compositions. c) ^87^Rb{^1^H} REDOR spectra of “Rb_0.03_FA_0.97_PbI_3_” with (*S*_1_) and without (*S*_0_) 3 ms of ^1^H dipolar recoupling. The difference spectrum is shown in purple. All spectra were acquired at room temperature and 20 kHz MAS. Experimental details are given in SI, together with a link to all the raw data.

Having shown that FA^+^ can substitute into the Rb‐based inorganic phase in bromide perovskite formulations, we now investigate whether this can also occur for iodide‐based perovskites. Mechanosynthesized samples with “Rb_*x*_FA_1−*x*_PbI_3_” compositions were prepared with *x* = 0.03 and 0.10. The XRD patterns show formation of the non‐perovskite δ‐RbPbI_3_ phase (Figure S8), analogous to observation of non‐perovskite FA_*y*_Rb_1−*y*_Pb_2_Br_5_ for the bromide samples (although the most stable Pb−X stoichiometry is different for the iodide and bromide phases). Figure 5a shows the ^87^Rb NMR spectra of the mechanosynthesized “Rb_*x*_FA_1−*x*_PbI_3_” samples and pure reference δ‐RbPbI_3_. The mixed compositions show peaks at 45.5 ppm which match pure δ‐RbPbI_3_ (although the 2^nd^ order quadrupolar features are smoothed out, see Figure S9 for the fitted quadrupolar parameters). Together with the XRD data, this indicates that Rb^+^ does not incorporate in FAPbI_3_, in line with previous studies.^[31]^

To probe the possibility of FA^+^ incorporation into the δ‐RbPbI_3_ phase, we carried out ^87^Rb→^1^H HETCOR and CP experiments for “Rb_0.03_FA_0.97_PbI_3_” and “Rb_0.10_FA_0.90_PbI_3_” samples (Figure 5b). As for the bromide samples, a CP signal is observed at a lower ^1^H shift (ca. 6.9 ppm), although the signal is weaker for the iodide samples. Again, the peak position of the ^87^Rb→^1^H signal does not match that of FA^+^ in FAPbI_3_ (i.e., the direct ^1^H and ^87^Rb→^1^H signals do not overlap), indicating that the Rb^+^ is not present in the FAPbI_3_ perovskite phase. Instead, this suggests substitution of FA^+^ into the non‐perovskite δ‐RbPbI_3_ phase, as seen for the bromide formulations.

This is confirmed by ^87^Rb{^1^H} REDOR experiments (Figure 5c and Figure S10). The “Rb_0.03_FA_0.97_PbI_3_” sample shows clear dephasing after 3 ms of recoupling, demonstrating atomic‐scale proximity between Rb^+^ and FA^+^. However, the dephasing is much slower than for the bromide samples (Figure S10), implying significantly less incorporation of FA^+^ into the iodide non‐perovskite phase. Although the low substitution level and poor sensitivity in the REDOR dephasing curves make quantitative analysis challenging, we estimate substitution of up to *y* ≈ 15 % in the δ‐FA_*y*_Rb_1−*y*_PbI_3_ phase (Figure S10) (See Supplementary Note 2 for the chemical equation of the formation reaction).

To understand the effect of the FA‐substituted non‐perovskite phase (δ‐FA_y_Rb_1−y_PbI_3_) on the optoelectronic properties of the perovskite phase, photoluminescence (PL) measurements were performed for α‐FAPbI_3_ and “Rb_0.03_FA_0.97_PbI_3_” thin‐film samples (Figure S11). As can be seen from the PL spectra, the “Rb_0.03_FA_0.97_PbI_3_” sample has higher PL intensity than the α‐FAPbI_3_ sample, indicating decreased non‐radiative recombination. This most likely arises due to increased crystallinity of the perovskite phase and/or passivation of surface defects.[^43^, ^56^] We propose that incorporation of FA^+^ into the non‐perovskite δ‐FA_*y*_Rb_1−*y*_PbI_3_ phase modulates the interaction with the perovskite phase and this is an important parameter to consider for the observed improvement in efficiency and stability of PSCs prepared with Rb^+^.

## Conclusions

We have determined the atomic‐level speciation and phase segregation in mechanosynthesized rubidium‐containing lead‐halide perovskite formulations. Notably, by using ^1^H−^87^Rb double resonance NMR experiments, we observe that instead of Rb^+^ incorporating in the perovskite lattice, FA^+^ incorporates into Rb‐based non‐perovskite phases. Up to ~60 % FA^+^ incorporation was observed in the inorganic Rb‐based phase for the bromide system, while a lesser extent of substitution (~15 %) was observed for the iodide containing system. In light of these results, we hypothesize that the substitution of FA^+^ into the non‐perovskite phase may contribute to the greater stability conferred by Rb salts in the synthesis of FA‐based perovskites.

## Conflict of Interests

The authors declare no conflict of interest.

1

## Supporting information

As a service to our authors and readers, this journal provides supporting information supplied by the authors. Such materials are peer reviewed and may be re‐organized for online delivery, but are not copy‐edited or typeset. Technical support issues arising from supporting information (other than missing files) should be addressed to the authors.

Supporting Information

## Data Availability

Raw and processed NMR, and XRD data are available at DOI: 10.5281/zenodo.13928501 with a CC‐BY‐4.0 (Creative Commons Attribution‐ShareAlike 4.0 International) license.
