# Supplementary material for: Formamidinium Incorporates into Rb‐based Non‐Perovskite Phases in Solar Cell Formulations
Source: Angew Chem Int Ed Engl. 2024 Nov 14;64(5):e202416938. doi: 10.1002/anie.202416938 (PMC11773310; doi:10.1002/anie.202416938)
Supplement: Supplementary file 1 — Supporting Information [file ANIE-64-e202416938-s001.pdf]

Angewandte Chemie
Eine Zeitschrift der Gesellschaft Deutscher Chemiker

Supporting Information

Formamidinium Incorporates into Rb-based Non-Perovskite Phases in Solar Cell Formulations

*U. Gunes, M. A. Hope, Y. Zhang, L. Zheng, L. Pfeifer, M. Grätzel, L. Emsley**

Supporting Information

Formamidinium Incorporates into Rb-based Non-perovskite Phases in Solar Cell Formulations

Ummugulsum Gunes[1], Michael A. Hope[1,2], Yuxuan Zhang[1], Likai Zheng[1], Lukas Pfeifer[1], Michael Grätzel[1], Lyndon Emsley*[1]

[1]Institut des Sciences et Ingenierie Chimiques, École Polytechnique Fedérale de Lausanne (EPFL), CH-1015 Lausanne, Switzerland
[2] Department of Chemistry, University of Warwick, Gibbet Hill Road, Coventry, CV4 7AL, UK

Raw and processed NMR, and XRD data are available at DOI:10.5281/zenodo.13928501 with a CC-BY-4.0 (Creative Commons Attribution-ShareAlike 4.0 International) license.

Experimental Section

Materials: The following materials used for the mechanosynthesis were purchased from Sigma Aldrich: PbI_2 (99%), $PbBr_2$ (99.999%), FAI (≥98%), FABr (≥98%), RbI (99.9%), RbBr (99.8%).

Bulk sample preparation: Perovskite samples were mechanosynthesized by grinding the appropriate molar ratios of the chemicals (Table S1, S2 and S3) in a ball mill (Retsch Ball Mill MM-200) for 30 min (1 h for $RbPb_2Br_5$ and $RbPbI_3$ samples) at 25 Hz using an agate jar and an agate ball (Ø 10 mm), followed by an annealing of the resulting powders at 150 °C for 15 min to mimic the thin-film fabrication process. The $FAPbBr_3$ sample was mechanosynthesized for 30 min at 25 Hz using an Eppendorf vial (2 mL) and a stainless-steel ball (Ø 4 mm) then the sample was annealed at 150 °C for 15 min.[1-2] The phases were identified by XRD (Bruker D8 Discover Vario) with Cu $K\alpha_1$ radiation (1.5406 Å). Pawley refinement was performed in Topas. The errors for the lattice parameters were estimated by changing each lattice parameter until the fit agreement became poor by eye.

Perovskite precursor solution preparation: The $FAPbI_3$ precursor solution was prepared by dissolving 1145 mg $FAPbI_3$ powder and 42.5 mg MACl into a mixed solvent containing 800 μL DMF and 200 μL DMSO. The "$Rb_{0.03}FA0_{.97}PbI_3$" precursor was prepared by adding 3 mol% of $RbPbI_3$ into the $FAPbI_3$ precursor.

Perovskite thin-film fabrication: The glass substrates were cleaned by subsequently rinsing in detergent, DI water, and ethanol with sonication for 15min, respectively. The perovskite precursor was spin-coated on top of the substrate at 6000 rpm for 50 s, 1 mL of diethyl ether was dropped on the substrate 10 s after the beginning of spin coating. Then, the substrate was transferred onto a hot plate, following by annealing at 150 °C for 10 min.

Solid-state NMR Spectroscopy

Solid-state MAS NMR spectra of ^1H and ^{87}Rb were performed at room temperature and 11.7 T using a Bruker Avance III spectrometer equipped with a 3.2 mm low-temperature CPMAS probe. All the experiments were run at room temperature (298 K sample temperature as measured by the sensor inside the stator) without active regulation. ^1H spectra were recorded using a Hahn-echo sequence and a recycle delay of 15 s. The spectra were referenced to adamantane at 1.85 ppm. ^{87}Rb spectra were recorded with a CT-selective Hahn echo with a single rotor period (per half echo) and with a recycle delay of 0.3 s. 20 kHz ^{87}Rb radiofrequency field amplitudes were used. The effective nutation frequency of CT-selective pulses for $I = 3/2$ ^{87}Rb is twice the specified value. A ^1H radiofrequency field amplitude of 100 kHz was used for REDOR recoupling. ^{87}Rb experiments were referenced to solid RbI at 177.1 ppm. $^{87}Rb \rightarrow {}^1H$ HETCOR experiments were performed at 20 kHz MAS, using a contact time of 5 ms with rf amplitudes of ~13.5 and ~55 kHz for ^{87}Rb and ^1H, respectively, and an effective recycle delay of 0.3 s. All the pulse sequences and complete acquisition parameters are given with the raw data.

Photoluminescence Spectroscopy

Photoluminescence measurement was performed using an Andor Kymera 193i spectrograph, and a 660 nm continuous-wave laser (OBIS, Coherent) set at 1-Sun equivalent photon flux (1.1 μm beam full-width half-maximum, 632 μW). PL spectra were collected at normal incidence using a 0.1 NA, 110 μm diameter optical fiber.

Table S1: Masses (mg) of the chemicals used for mechanosynthesis of "$Rb_xFA_{1-x}PbBr_3$" formulations.

x	*0*	*0.01*	*0.03*	*0.10*
FABr	50.8	51.0	50.3	45.7
RbBr	-	0.7	2.0	6.6
$PbBr_2$	149.2	149.2	149.0	148.0

Table S2: Masses (mg) of the chemicals used for mechanosynthesis of $RbPb_2Br_5$.

RbBr	36.8
$PbBr_2$	163.4

Table S3: Masses (mg) of the chemicals used for mechanosynthesis of "$Rb_xFA_{1-x}PbI_3$" formulations and $RbPbI_3$.

x	*0.03*	*0.10*	*1.00*
FAI	52.4	48.9	-
RbI	1.9	6.6	63.0
PbI_2	145.7	144.6	136.6

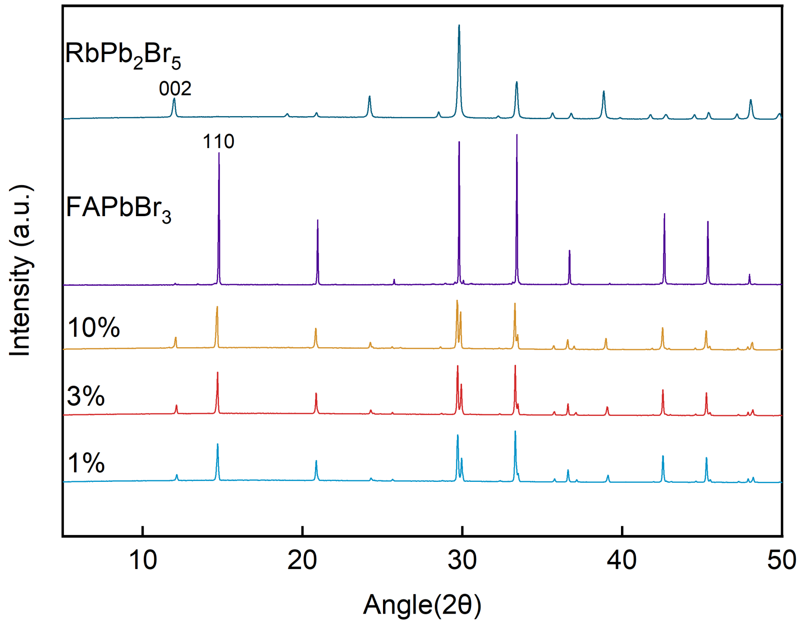

Figure S1. XRD patterns of the mechanosynthesized "$Rb_xFA_{1-x}PbBr_3$" formulations (x = 0.01, 0.03 and 0.10), $RbPb_2Br_5$ and $FAPbBr_3$ samples. Miller indices are shown for the first reflections in the reference compounds.

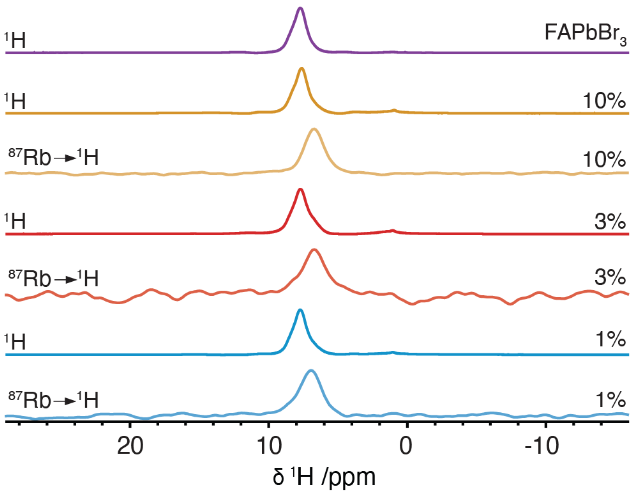

Figure S2. ^{1}H spectra of $FAPbBr_3$ and "$Rb_xFA_{1-x}PbBr_3$" formulations (x = 1%, 3%, 10%), ^{87}Rb→^{1}H CP spectra of the "$Rb_{0.01}FA_{0.99}PbBr_3$" and "$Rb_{0.03}FA_{0.97}PbBr_3$" compositions, and a slice from the ^{87}Rb→^{1}H HETCOR spectrum of the "$Rb_{0.10}FA_{0.90}PbBr_3$" sample.

Table S4: Lattice parameters of the mechanosynthesized "$Rb_xFA_{1-x}PbBr_3$" formulations obtained from Pawley refinement, as well as pure $FAPbBr_3$ and $RbPb_2Br_5$. The bracketed number indicates the error for the 3rd decimal place.

x	*FAPbBr₃*	*RbPb₂Br₅ (a)*	*RbPb₂Br₅ (c)*
Pure	5.994	8.440	14.579
1%	5.995(2)	8.436(6)	14.478(4)
3%	5.995(3)	8.439(4)	14.497(6)
10%	5.993(4)	8.436(4)	14.523(7)

Table S5: Relative lattice parameters (%) of the mechanosynthesized "$Rb_xFA_{1-x}PbBr_3$" formulations obtained from Pawley refinement. The relative change is defined as $(\lambda_{\mathrm{mixed}} - \lambda_{\mathrm{pure}})/\lambda_{\mathrm{pure}}$, where λ_{mixed} is the lattice parameter in the mixed formulation and λ_{pure} is the lattice parameter in $FAPbBr_3$ or $RbPb_2Br_5$.

x	*FAPbBr₃*	*RbPb₂Br₅ (a)*	*RbPb₂Br₅ (c)*
1%	0.03 ± 0.05	-0.05 ± 0.10	-0.69 ± 0.04
3%	0.02 ± 0.07	-0.01 ± 0.07	-0.57 ± 0.06
10%	-0.01 ± 0.08	-0.04 ± 0.06	-0.39 ± 0.07

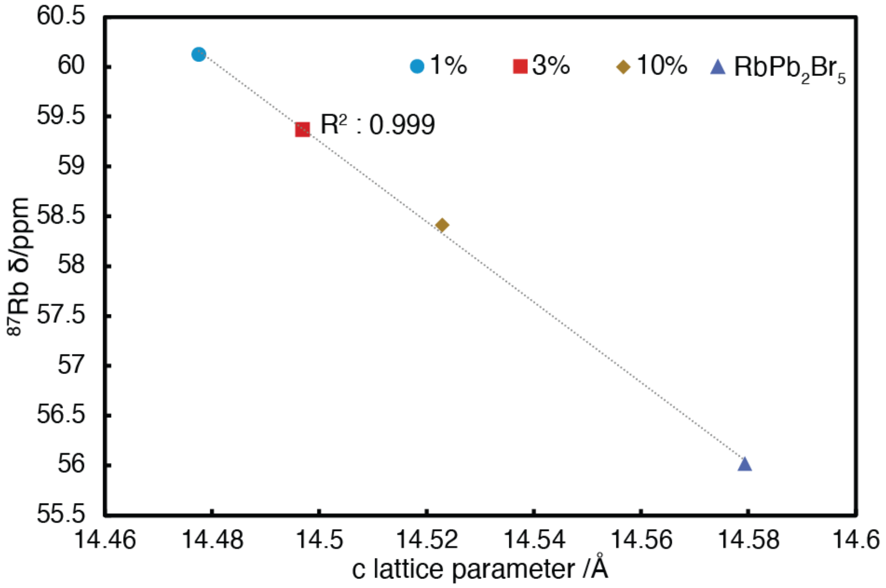

Figure S3. Linear regression of the ^{87}Rb chemical shifts against the *c* lattice parameter obtained from Pawley refinement for the $FA_yRb_{1-y}Pb_2Br_5$ phase in "$Rb_xFA_{1-x}PbBr_3$" formulations (x: 0.01, 0.03, 0.10).

Supplementary Note 1: Analysis of REDOR Dephasing

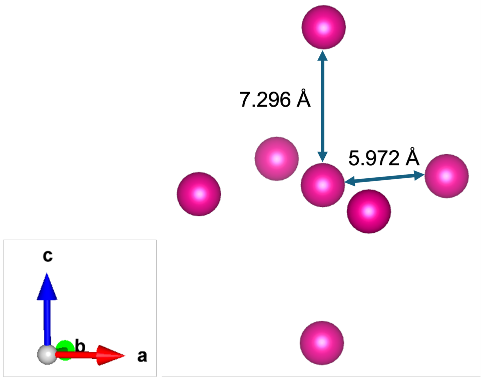

Figure S4: Distances between neighbouring Rb cations in the $RbPb_2Br_5$ crystal structure.

To estimate the FA^+ content in the $FA_yRb_{1-y}Pb_2Br_5$ phase, we simulated the $^{87}Rb\{^1H\}$ REDOR curves. As shown in Figure S4, each Rb has four nearest neighbours in the *ab* plane (5.972 Å) and two neighbours above and below in the *c* direction (7.296 Å). We considered three possible orientations of FA^+ within the structure, assuming that the FA^+ axes align with the Rb–Rb directions (Figure S5). We also considered a rotationally averaged model where all five protons are effectively located at the centre of mass of the molecule.

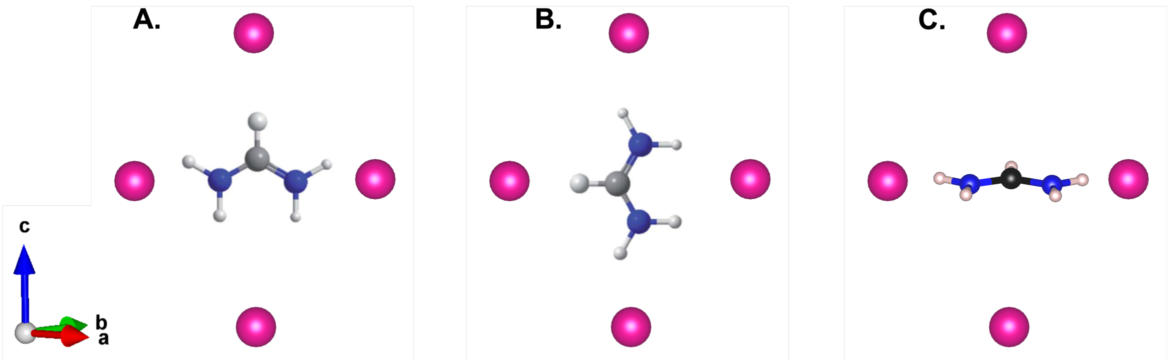

Figure S5: Considered orientations of FA^+ in the $RbPb_2Br_5$ crystal structure. The Rb atoms located in the front and the back are omitted for clarity.

For each orientation, we calculated the ^1H–^{87}Rb distance for each proton in FA^+ and hence the ^1H–^{87}Rb dipolar coupling. We then simulated the $^{87}Rb\{^1H\}$ REDOR dephasing curves for a single ^1H–^{87}Rb spin pair at each possible distance using SIMPSON.[3] To simulate the overall dephasing curve, we considered a central ^{87}Rb atom and calculated the probability of having FA^+ substitute for Rb in each of the six nearest neighbour sites (Figure S4). For each possible $[RbRb_zFA_{6-z}]$ configuration, the REDOR dephasing curve was assumed to be given by the product of the dephasing curves for each ^1H–^{87}Rb spin pair in that configuration (note that this does not allow for dipolar oscillations, but these are not significant given the relatively short recoupling times, and the averaging effect of random substitution). The total simulated curve is then the sum of the dephasing curves for each configuration, weighted by its probability. Finally, the FA^+ content was optimized, thus changing the probability of each configuration, to best fit the experimental REDOR data for all the experimental samples.

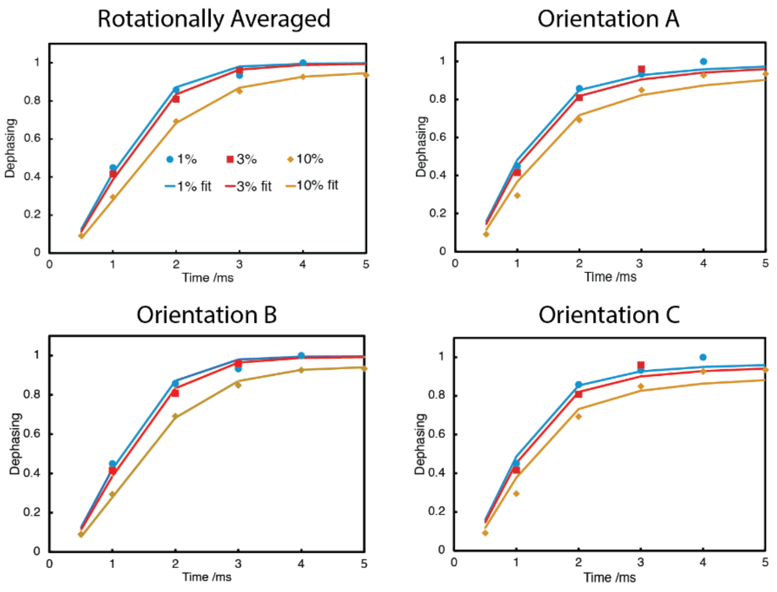

Figure S6. Simulated REDOR dephasing curves of $FA_yRb_{1-y}Pb_2Br_5$ phases as a function of recoupling time with different FA^+ orientations. The y values were fitted to best match the experimental data for "$Rb_xFA_{1-x}PbBr_3$" formulations with $x = 0.01, 0.03, 0.10$ (values given in Table S6)

Figure S6 shows the best-fit simulated $^{87}Rb\{^1H\}$ REDOR curves for each of the four models considered. To assess the models, the agreement between the simulated and experimental data was quantified by the χ^2, and the fitted FA^+ content was plotted against the experimental *c* lattice parameter (Figure S7) and the R^2 calculated (since a linear correlation is predicted by Vegard's law). As shown in Table S5, the best agreement with both the REDOR data and Vegard's law is obtained for orientation B, where the N–N direction is aligned with *c* axis. This may be because the cavity is larger in the *c* direction than in the *ab* plane. Consequently, the estimated FA^+ contents for orientation B are reported in the main text. We note that the rotationally averaged model gives similar results to those of orientation B.

Table S6: FA^+ content, *y*, for the $FA_yRb_{1-y}Pb_2Br_5$ phase in "$Rb_xFA_{1-x}PbBr_3$" formulations (*x*: 0.01, 0.03, 0.10), calculated by fitting the REDOR dephasing curves, assuming different orientations of FA^+ within the structure (Figure S5). For each orientation, the agreement with the REDOR data was quantified by $\chi^2 = \sum(\text{expt.} - \text{calc.})^2$, summed over all the datapoints for all three samples. To quantify the agreement with Vegard's law, the fitted FA^+ contents for each orientation were plotted against the experimental *c* lattice parameter and the R^2 calculated.

**FA^+ Content (*y*)**	Rotationally averaged	Orientation A	Orientation B	Orientation C
$RbPb_2Br_5$	0	0	0	0
10% Rb	0.42	0.35	0.38	0.33
3% Rb	0.61	0.45	0.56	0.41
1% Rb	0.69	0.49	0.63	0.45
Vegard's law R^2	0.992	0.97	0.993	0.966
REDOR χ^2	0.0058	0.0183	0.0056	0.0254

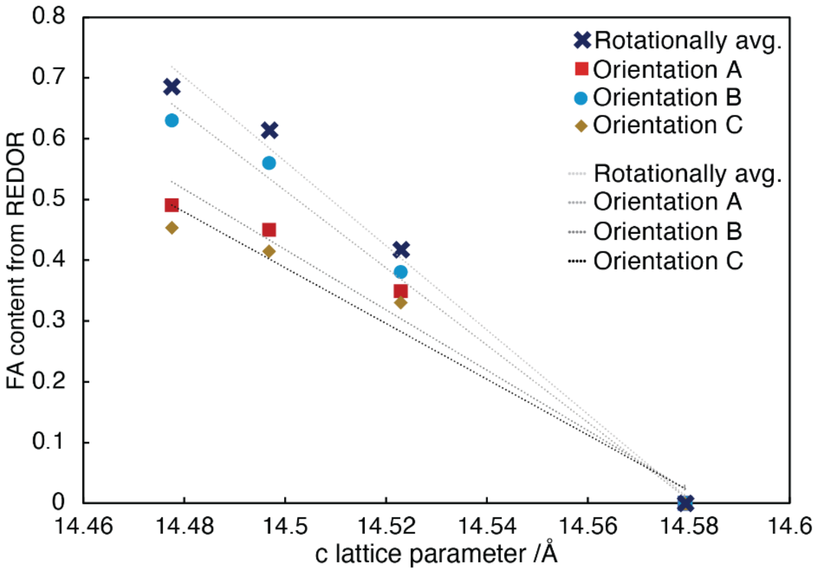

Figure S7. Linear regressions between the estimated FA$^+$ contents of the FA$_y$Rb$_{1-y}$Pb$_2$Br$_5$ phase in "Rb$_x$FA$_{1-x}$PbBr$_3$" formulations obtained from the REDOR simulations for different possible FA$^+$ orientations and the experimental *c* lattice parameters obtained from Pawley refinement.

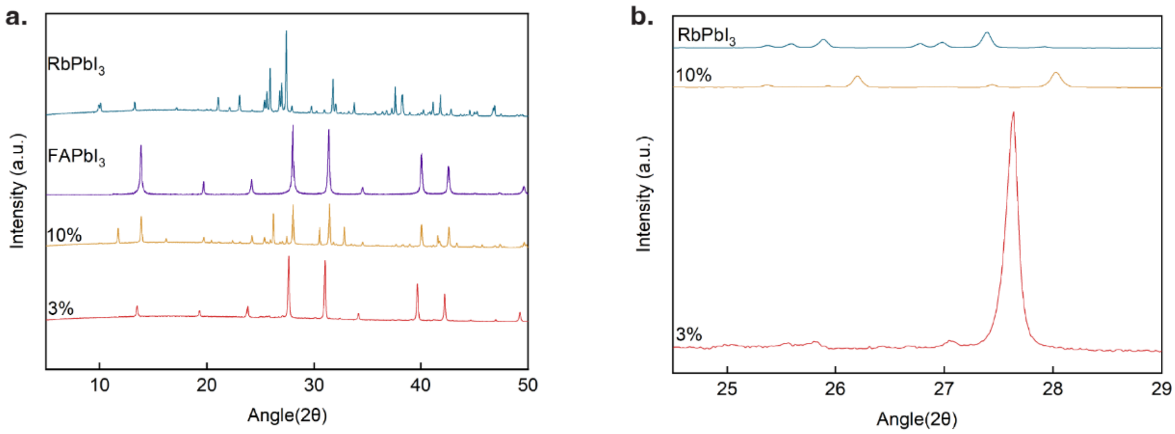

Figure S8. **a** XRD patterns of the mechanosynthesized "Rb$_x$FA$_{1-x}$PbI$_3$" formulations ($x = 0.03$ and 0.10), FAPbI$_3$ and pure δ-RbPbI$_3$. **b** XRD patterns of the mechanosynthesized "Rb$_x$FA$_{1-x}$PbI$_3$" formulations ($x = 0.03$ and 0.10) and pure δ-RbPbI$_3$, the region between 24.5°-29° is zoomed to show the characteristics peaks of δ-RbPbI$_3$ in each sample.

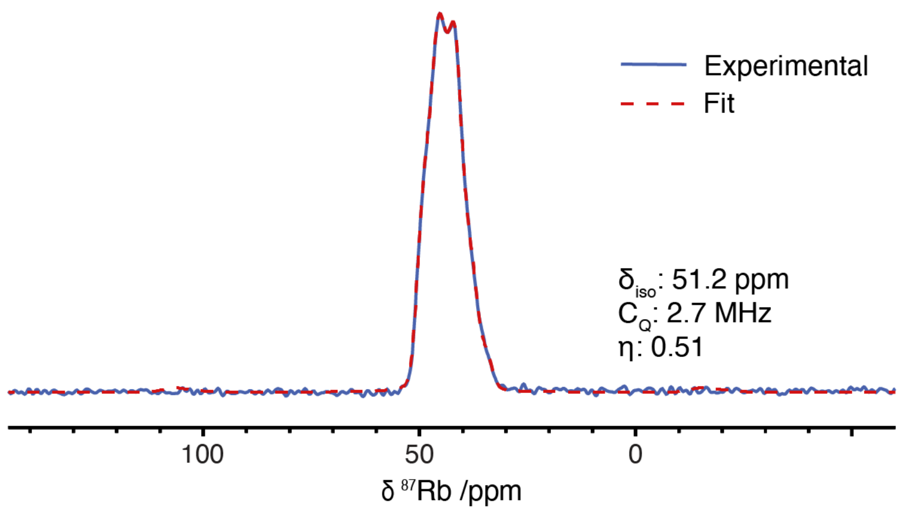

Figure S9. Fitted ^{87}Rb NMR spectrum of the δ-$RbPbI_3$ sample. The spectrum is fitted by using the Solid Lineshape Analysis (sola) package in TopSpin. The spectrum shows a 2nd order quadrupolar lineshape due to the fact that ^{87}Rb is a quadrupolar nucleus ($I = 3/2$). Inset: Fitted ^{87}Rb NMR parameters of δ-$RbPbI_3$.

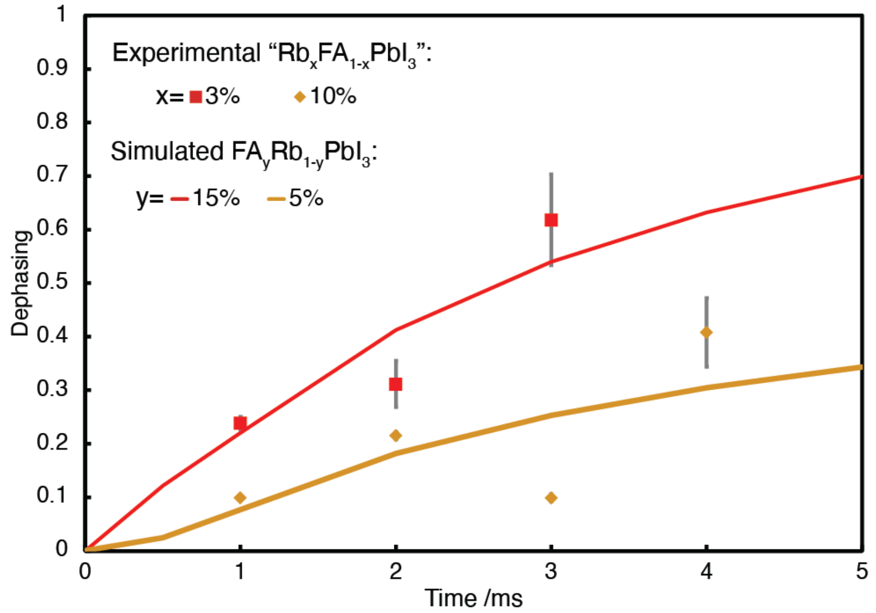

Figure S10. $^{87}Rb\{^1H\}$ REDOR dephasing, $(S_0 - S_1)/S_0$, as a function of the recoupling time (points) for the δ-$FA_yRb_{1-y}PbI_3$ phases in "$Rb_xFA_{1-x}PbI_3$" compositions (x: 0.03, 0.10), and simulated dephasing curves (lines) for optimized FA^+ contents. Due to the greater complexity of the $RbPbI_3$ structure, an average orientation of FA^+ was considered, i.e., all five ^1H nuclei are assumed to have an average position at the centre of the cation site. Given the low sensitivity of the experimental data, this assumption is not expected to add a significant additional error to the estimation of y.

Supplementary Note 2: Chemical equations of the formation reactions for the "$Rb_xFA_{1-x}PbBr_3$" and "$Rb_xFA_{1-x}PbI_3$" formulations

The balanced chemical reactions for the mechanochemical reaction of the precursors to form the perovskite and non-perovskite phases can be written as follows. x indicates the amount of Rb^+ included in the formulation, y is the amount of FA^+ incorporated in the non-perovskite phase, and a gives the proportions of the perovskite and non-perovskite phases. Note that for the bromide case, owing to the different Pb:Br ratios in the perovskite and non-perovskite phases, there must be some unreacted FABr to balance the equation. The unreacted FABr cannot be resolved from the major perovskite peak in the ^{1}H NMR experiments. In addition, due to the weak diffraction of FABr, it also was not detected in the XRD measurements.

$$(1-x)\mathrm{FABr} + x\,\mathrm{RbBr} + \mathrm{PbBr_2} \rightarrow (1-a)\,\mathrm{FAPbBr_3} + a\,\mathrm{FA_yRb_{1-y}Pb_2Br_5} + a\,\mathrm{FABr}$$

$$(1-x)\mathrm{FAI} + x\,\mathrm{RbI} + \mathrm{PbI_2} \rightarrow (1-a)\,\mathrm{FAPbI_3} + a\,\delta\text{-}\mathrm{FA_yRb_{1-y}PbI_3}$$

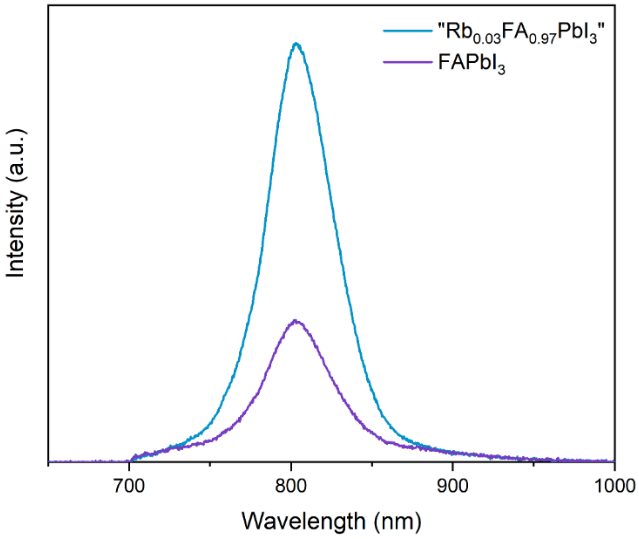

Figure S11. PL spectra of α-$FAPbI_3$ and "$Rb_{0.03}FA_{0.97}PbI_3$" samples (both samples contain MACl as an additive).

Reference

[1] A. Mishra, M. A. Hope, M. Grätzel, L. Emsley, *J. Am. Chem. Soc.* **2023**, *145*, 978-990.

[2] D. Bi, X. Li, J. V. Milić, D. J. Kubicki, N. Pellet, J. Luo, T. LaGrange, P. Mettraux, L. Emsley, S. M. Zakeeruddin, M. Grätzel, *Nat.Commun.* **2018**, *9*, 4482.

[3] M. Bak, J. T. Rasmussen, N. C. Nielsen, *J. Magn. Reson.* **2000**, *147*, 296-330.
